# Local and global distensibility assessment of abdominal aortic aneurysms *in vivo* from probe tracked 2D ultrasound images

**DOI:** 10.3389/fmedt.2022.1052213

**Published:** 2023-01-06

**Authors:** Larissa C. Jansen, Hans-Martin Schwab, Frans N. van de Vosse, Marc R. H. M. van Sambeek, Richard G. P. Lopata

**Affiliations:** ^1^ Photoacoustics and Ultrasound Laboratory Eindhoven (PULS/e), Department of Biomedical Engineering, Eindhoven University of Technology, Eindhoven, Netherlands; ^2^ Department of Vascular Surgery, Catharina Hospital Eindhoven, Eindhoven, Netherlands; ^3^ Cardiovascular Biomechanics, Department of Biomedical Engineering, Eindhoven University of Technology, Eindhoven, Netherlands

**Keywords:** distensibility, cardiovascular, patient specific geometries, ultrasound imaging, abdominal aortic aneurysm (AAA)

## Abstract

Rupture risk estimation of abdominal aortic aneurysm (AAA) patients is currently based on the maximum diameter of the AAA. Mechanical properties that characterize the mechanical state of the vessel may serve as a better rupture risk predictor. Non-electrocardiogram-gated (non-ECG-gated) freehand 2D ultrasound imaging is a fast approach from which a reconstructed volumetric image of the aorta can be obtained. From this 3D image, the geometry, volume, and maximum diameter can be obtained. The distortion caused by the pulsatility of the vessel during the acquisition is usually neglected, while it could provide additional quantitative parameters of the vessel wall. In this study, a framework was established to semi-automatically segment probe tracked images of healthy aortas (*N* = 10) and AAAs (*N* = 16), after which patient-specific geometries of the vessel at end diastole (ED), end systole (ES), and at the mean arterial pressure (MAP) state were automatically assessed using heart frequency detection and envelope detection. After registration AAA geometries were compared to the gold standard computed tomography (CT). Local mechanical properties, i.e., compliance, distensibility and circumferential strain, were computed from the assessed ED and ES geometries for healthy aortas and AAAs, and by using measured brachial pulse pressure values. Globally, volume, compliance, and distensibility were computed. Geometries were in good agreement with CT geometries, with a median similarity index and interquartile range of 0.91 [0.90–0.92] and mean Hausdorff distance and interquartile range of 4.7 [3.9–5.6] mm. As expected, distensibility (Healthy aortas: 80 ± 15·10^−3^ kPa^−1^; AAAs: 29 ± 9.6·10^−3^ kPa^−1^) and circumferential strain (Healthy aortas: 0.25 ± 0.03; AAAs: 0.15 ± 0.03) were larger in healthy vessels compared to AAAs. Circumferential strain values were in accordance with literature. Global healthy aorta distensibility was significantly different from AAAs, as was demonstrated with a Wilcoxon test (*p*-value = 2·10^−5^). Improved image contrast and lateral resolution could help to further improve segmentation to improve mechanical characterization. The presented work has demonstrated how besides accurate geometrical assessment freehand 2D ultrasound imaging is a promising tool for additional mechanical property characterization of AAAs.

## Introduction

Patients with an abdominal aortic aneurysm (AAA), a local dilatation of the abdominal aorta, are at risk for aneurysm rupture. Currently, this risk is considered high when the diameter reaches a threshold of 5.0 cm for females or 5.5 cm for males, or when the diameter growth exceeds a threshold of 1.0 cm per year (1, 2). However, previous studies have shown that AAAs can rupture before reaching the diameter threshold or can remain stable after exceeding the threshold (3–5). From a mechanical perspective, the vessel wall will rupture once the stress on the wall exceeds the wall strength. Therefore, there is a need for a more patient specific approach that includes mechanical characterization.

Finite element models are tools that can be used to estimate the mechanical state of the vessel during patient follow-up, and to model growth and remodeling of the vascular tissue. Different mechanical properties such as vessel wall stress, strain, and shear modulus can be assessed indirectly (6, 7). However, modeling AAAs adequately requires patient specific information on the shape and material properties of the vessel. Computed tomography (CT) is considered the gold standard to extract the patient specific geometry. This imaging technique, however, involves the use of ionizing radiation and nephrotoxic contrast agent. Furthermore, it typically lacks temporal information. Alternatively, Magnetic Resonance Imaging (MRI) could be used to extract both the geometry and dynamic information. However, MRI has high costs and long scanning times. Ultrasound (US) imaging is an imaging technique which is considered safe, has low costs, and can easily be used at the patient's bedside. An additional advantage of time resolved US imaging is the availability of temporal information, from which mechanical properties can be assessed. These properties can be used to personalize mechanical models, rather than using properties from literature (6, 7). Examples of these properties are wall strain, compliance, and distensibility. Compliance measures the ability of the vessel to increase and decrease in volume resulting from a change in intravascular pressure (8). Distensibility captures this volume change and takes the initial size of the vessel into account (9).

One way to assess distensibility was by evaluating the diameter change at the maximal diameter location (10). However, this approach lacks characterization of the complete vessel. Alternatively, maximum mean segmental dilatation has been assessed using 2D tissue doppler imaging in a single longitudinal imaging plane, from which segmental compliance and distensibility were computed (11, 12). Although a vessel segment, rather than a single location of the vessel is evaluated with this approach, compliance and distensibility computed from diameter change along a single line assumes the vessel to be a circle. Specifically in aneurysms, this is not a valid assumption. Hence, an area or volume-based computation would be more accurate.

With the development of matrix arrays for 3D US imaging, the aneurysm volume can now be assessed (13). Moreover, with time-resolved 3D US, vessel dimension changes during the cardiac cycle can be captured (6). However, the main disadvantages of this imaging approach are the limited temporal resolution, field of view, and image quality. Another 3D approach is freehand 2D US with 3D image reconstruction, where an operator moves a 2D US transducer, that is connected to a probe tracker, after which a single 3D volume is reconstructed offline. Compared to imaging with a matrix array, this approach has lower costs, higher volume quality and an improved field of view (14–17). So far, with electrocardiogram-gated (ECG-gated) or non-ECG-gated freehand 2D US of AAAs, the patient specific geometry and volume have been assessed, from which the vessel diameter and area at the maximal aneurysm diameter location have been computed (14, 15, 17–19). It was previously demonstrated that reproducible AAA volume measurements can be obtained from 3D reconstructions obtained with freehand 2D US (17). Furthermore, it has been shown that AAA volumes measured from volumetric images that were reconstructed from freehand 2D US are comparable to those obtained with computed tomography angiography (CTA) (14). However, registration with CT geometries and quantification of similarity and overlap between the geometries has not been demonstrated yet.

What is often neglected with non-ECG-gated freehand 2D US of AAAs is the pulsatile motion of the vessel, even though it distorts volumetric image reconstruction and volume computation. ECG gating has been used to obtain a volume in the same phase of the cardiac cycle (18, 19). However, the acquisition time is then extended from seconds to minutes and the measurement becomes more susceptible to patient motion. In a freehand 2D US study on carotids, it was demonstrated how heart frequency detection and filtering can correct for the pulsatile motion by considering the individual frames, rather than the reconstructed volumetric image (20). In this way, mean arterial pressure (MAP) state geometries of carotid arteries could be obtained. Since the pulsatility, that is captured during the acquisition, can be filtered out, it could also be exploited for estimation of local vessel properties, such as circumferential strain and in combination with a pulse pressure measurement, distensibility or pressure modulus.

In this study, distensibility of healthy abdominal aortas and AAAs was locally and globally assessed from fast, non-EGC-gated, freehand 2D US acquisitions and non-invasive blood-pressure measurements. A semi-automatic segmentation and automatic spatiotemporal signal processing framework were developed to obtain the vessel geometry at end diastole (ED), end systole (ES) and MAP. Distensibility and mean circumferential strain were estimated locally by evaluating area changes, and distensibility was assessed globally from volume changes. Feasibility of this approach was evaluated by registration and comparison of aneurysm geometries with geometries obtained from the gold standard CT. To evaluate if our approach is sensitive to different levels of distension, mechanical properties of healthy aortas and AAAs were assessed, and compared to previous studies.

## Materials and methods

### Study population

This study was approved by the local medical ethics committee of the Catharina Hospital, Eindhoven (NL). Young healthy volunteers (*N* = 12) and AAA patients (*N* = 26) participated in this study after giving their written, informed consent. After initial review of the US data, 2 healthy volunteer and 7 AAA datasets were excluded due to poor image quality, and 3 AAA datasets were excluded due to excessive motion as a result of breathing during the US measurement. The remaining AAA patients (*n* = 16, age range: 58–90 years) were grouped into two categories. The patients of the first group (*n* = 9) had undergone a CTA scan as part of regular clinical practice, whereas the second group (*n* = 9) consisted of patients that underwent a brachial blood pressure (BP) measurement prior to the US acquisition. Two patients were included in both groups, since both brachial BP and a CTA scan were available. The healthy volunteers (*N* = 10, age range: of 24–29 years) underwent a brachial BP measurement besides the US acquisitions. The age, brachial BP values and AAA maximal diameter are summarized in Table 1.

**Table 1:** Summary of the age, gender (female/male), brachial diastolic BP (p_dia_) and systolic BP (p_sys_) for all 10 healthy volunteers (V1–V10) and 16 patients.

	Age (years)	F/M	BP (mmHg)			Age (years)	F/M	D_max_ (mm)	BP (mmHg)			Age (years)	F/M	D_max_ (mm)	BP (mmHg)	
			P_dia_	P_sys_					P_dia_	P_sys_					P_dia_	P_sys_
V1	25	M	68	115	A1	75	F	52	-	-	B1	90	F	52	152	96
V2	27	M	79	126	A2*	80	M	55	163	93	B2	78	F	39	102	71
V3	25	F	78	110	A3	84	M	57	-	-	B3	72	M	52	112	68
V4	28	M	81	118	A4	73	M	56	-	-	B4	58	M	41	135	78
V5	29	F	81	107	A5	73	M	54	-	-	B5	74	M	40	136	91
V6	26	M	60	110	A6	79	M	56	-	-	B6	74	M	45	141	87
V7	25	M	69	118	A7	72	M	86	-	-	B7*	80	M	55	163	93
V8	24	F	64	110	A8	76	M	60	-	-	B8	83	M	48	165	87
V9	26	F	57	102	A9**	69	M	51	180	94	B9**	69	M	51	180	94
V10	25	F	55	108												
µV	26	-	69	112	µA	76	-	59	-	-	µB	75	-	47	143	85

The patients were divided into two groups, based on the types of measurements available. Patients of group A (A1–A9) underwent a CTA scan. Patients of group B (B1–B9) underwent a brachial BP measurement. Patient A2 and A9 belong to both categories indicated by one or two asterisks. Per group, the mean (*µ*) values are shown.

### Data collection

#### Brachial blood pressure measurement

Prior to the US measurement, the diastolic and systolic BP were measured with an arm-cuff while the subject was in supine position. van ‘t Veer et al. showed that brachial cuff pressure measurements overestimate the diastolic BP (P_dia_) by 12% and underestimate the systolic BP (P_sys_) by 5% compared with direct intra-aortic pressures (9). Hence the measured brachial cuff measurements were corrected accordingly.

#### Freehand 2D ultrasound imaging

**Figure 1:**
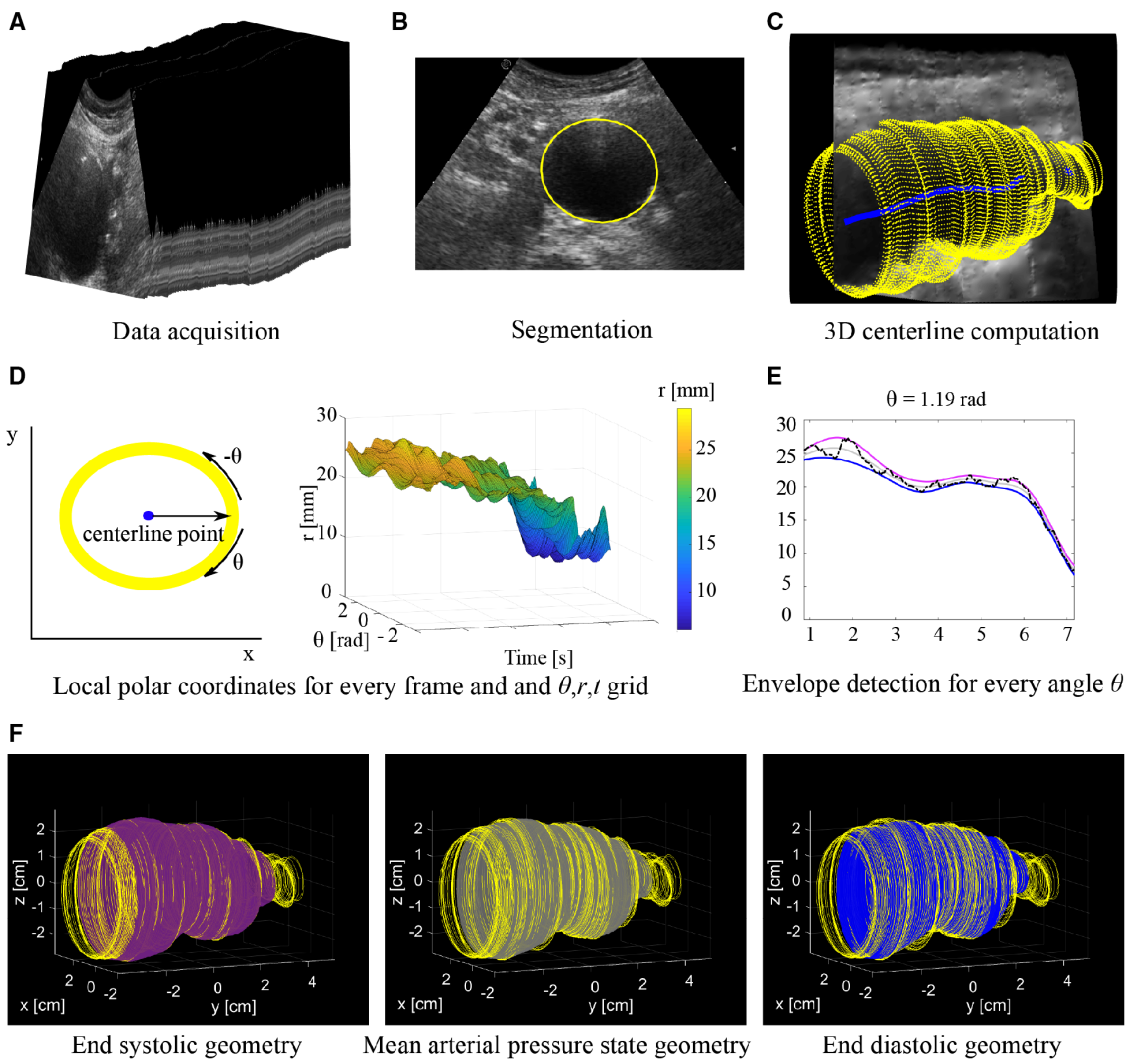
Overview of workflow. (**A**) Series of 2D US frames acquired with freehand 2D US imaging. (**B**) Segmentation is performed on the individual 2D US frames. (**C**) Contours segmented from each frame (yellow) are mapped into 3D space together with the 3D centerline (blue). (**D**) Conversion of coordinates of each frame to a local polar coordinate system centered around the center line and generating a *θ*, *r*, *t* grid. (**E**) Envelope detection on a radius time signal (black line) for a single angle *θ*. The radii at end diastole (blue line) and end systole (purple line) are estimated from which the radii at the mean arterial pressure state (grey line) are derived. (**F**) Final 3D geometries with from left to right: End systole (purple), mean arterial pressure state (grey) and end diastole (blue). Geometries are shown together with the original contours (yellow).

A series of 2D US images of the abdominal aorta were acquired while moving a CA431 2D curved array probe (center frequency: 2.6 MHz) connected to a commercial Esaote Mylab70.2 US system (Esaote Europe, Maastricht, the Netherlands) manually on the abdomen (Figure 1A). The entire acquisition was performed during breath-hold, while the subject was in supine position. As the measurement was performed freehand, the length of the acquisition and distance covered by the probe varied from measurement to measurement. The probe was connected to an electromagnetic probe tracking device (Curefab, Munich, Germany), which recorded the 3D probe orientation and a time stamp for every acquired image during the acquisition. The probe tracker was calibrated prior to usage. A study by Feurer et al. demonstrated that this probe tracker has a satisfactory reliability and accuracy (21). Their study showed a mean point accuracy of 1.52 mm and mean total error of distance measurements of 0.9%. Data was stored at a 25 Hz sampling rate. As the measurement was performed freehand, the length of the acquisition and distance covered by the probe varied from measurement to measurement. Consequently, the distance between samples varied per subject, depending on the speed of the probe.

#### Post-processing computed tomography datasets

CTA scans were acquired within 1 month (Table 2) from the US acquisition as part of regular clinical practice using a 256 slice CT scanner (Philips Healthcare, Best, the Netherlands), with a slice thickness of 3 mm. Hemodyn post-processing software (Philips Medical System and Eindhoven University of technology, the Netherlands) was used to semi-automatically obtain the 3D geometries of the aneurysm (7). With this software, the lumen-wall interface was segmented using a 3D active contour. In case intraluminal thrombus was present, the thrombus-wall interface and lumen-wall interface were segmented. Small manual adaptations were made after the segmentation process.

**Table 2:** Similarity indices and median hausdorff distances with interquartile range (IQR) of AAA geometries assessed from freehand 2D US data that were registered and compared to CT geometries.

Subject	Similarity index (−)	HD (mm), median (IQR)	Time between US and CT (days)
A1	0.88	6.6 [5.7–8.4]	20
A2	0.93	3.2 [2.8–3.8]	1
A3	0.91	4.5 [4.5–4.8]	11
A4	0.91	4.1 [3.6–4.8]	6
A5	0.93	3.0 [2.2–4.0]	2
A6	0.91	5.2 [4.4–6.8]	6
A7	0.90	7.3 [6.7–8.2]	2
A8	0.91	5.1 [4.5–6.0]	31
A9	0.90	4.7 [3.8–5.6]	27
median + IQ range	0.91 [0.90–0.92]	4.7 [3.9–5.6]	

### Geometry assessment

#### Segmentation of transverse 2D US images

Segmentation was performed to extract the lumen-wall interface of the aorta in the transverse images of the freehand acquisition. In the case intraluminal thrombus was present in AAAs, the thrombus-wall interface was segmented. Prior to segmentation, a Euclidean shortening flow filter, a well-known edge preserving de-speckling filter (22), was applied followed by a Gaussian filter with a 3 by 3 kernel size. These filters were applied to enhance the contrast between the wall and lumen region by reducing noise. Next, lumen-wall or thrombus-wall interface contours were segmented semi-automatically using an in-house toolbox, based on a star-Kalman approach by Guerrero et al. (23) that was adapted by de Ruijter et al. (20) and implemented in MATLAB (R2019b, Mathworks Inc., Natick, MA, USA). In this algorithm the lumen-wall or thrombus-wall interface is approximated by an ellipse-shape. The segmentation algorithm was initialized by manually defining an ellipse at the aorta location in the first frame. Next, the star algorithm was used to find the interface in the next frame. For this algorithm, a search region was defined around the ellipse of the previous frame. Within this search region the best edge positions, representing the lumen-wall or thrombus-wall interface positions, in the next frame were detected using step edge detection (24). The search region was extruded both inwards and outwards with respect to the ellipse, resulting in a radial thickness of 2.5 and 7 mm for healthy aortas and AAAs respectively. A larger search region was used for AAAs compared to healthy aortas, as besides geometry change due to pulsation, morphological vessel shape changes can occur from one frame to the next. Next, an ellipse was fitted through the high probability edge positions found in this search region, which was the final segmentation for this frame as well as the starting contour for a new search of the vessel wall in the next frame (Figure 1B). Using a Kalman filter, adapted from Guerrero et al., the frame-to-frame ellipse estimates were stabilized (20, 23).

#### 3D centerline detection

A 3D centerline was computed based on the centers of mass of the contours of the ED frames, which were automatically detected using heart frequency analysis. A heart frequency range was automatically detected from the artery area-time signal in the Fourier domain. The area of the lumen was obtained by converting the segmented contours into a binary mask, from which the lumen area was extracted using the pixel dimensions. Next, the area per frame signal was resampled to have equidistant time steps. The heart frequency range was then detected from the power-density spectrum of this signal, where it was defined as the highest peak in the physiological range of the power-density spectrum ± 0.2 Hz, allowing for a ± 12 beats per minute change throughout the acquisition. Next, the smallest radii in the signal separated by a period range corresponding to the detected heart frequency range were detected. Then, the signal was converted back to the original sampling in time and the frames closest to the timepoints of the smallest radii were defined as the ED frames. Next, the segmented contours of each ED frame were mapped from the local image coordinate system to the 3D Cartesian coordinate system according to the probe tracker orientation data. Finally, the 3D centerline (Figure 1C) was generated using 3D spline interpolation between the ellipse center points of the ED frame contours. A moving average filter with a kernel size of 15 samples was applied to the centerline, to smooth the centerline. Centerline coordinates were obtained for the remaining frames by computing the intersection of the centerline with the image planes of each frame.

#### End diastolic, end systolic and mean arterial pressure state geometry assessment

To obtain estimates of the ED, ES and MAP geometries, the contours were locally converted into the polar coordinate system (*r*, *θ*) with the local centerline location as the origin (Figure 1D). Then for each contour, the *r*, *θ* contour points were resampled to have equidistant angle intervals *θ*. Next, the *r*, *θ* coordinates of all contours were combined in a 3D grid with time *t* as the third dimension (*r*, *θ*, *t*). This was followed by envelope detection on the *r*-*t* signal for each angle *θ* individually to obtain radii at ES, *r_es_*. Next, the *r-t* signal was reversed by multiplying it with −1. Envelope detection on this signal provided the radii at ED, *r_ed_* (Figure 1E). The envelope detection was constrained such that only peaks separated by at least the minimal period of the heart frequency range were detected. The radii at MAP, *r_map_*, were assessed for each angle *θ* from the previously assessed *r_ed_* and *r_es_* according to:

(1) $$r_{map}=0.5\;(r_{es}-\;r_{ed})+r_{ed}$$

As the interpolation of the *r*-*t* signals were performed for each angle *θ* individually, local irregularities may occur. Hence, ellipses were fitted to the *θ-r* coordinates for every contour, after which the coordinates were transferred back to image coordinate system followed by mapping the newly obtained contours to 3D space (Figure 1F).

### Comparison with computed tomography

#### Registration

To register an US geometry with a CT geometry, the optimal translation and rotation that maximizes the overall similarity between the point clouds was found using an iterative closest point (ICP) algorithm (25, 26). An ICP algorithm minimizes the distances between two 3D point clouds according to the minimal distance difference principle. This is followed by computation of the global rotation and translation that aligns the two point clouds. An optimum is provided in result of an iterative process that runs until the root mean square error is under a set threshold, or when a set number of iterations is completed. In this study a publicly available ICP algorithm (compatible with MATLAB) was used for the registration process (27). Upon visual inspection of the results, the number of iterations was set to 30. Next the contours of the US data are transformed to the new registered position by rigid transformation of the coordinates using the global rotation and translation that was previously computed.

#### Comparison metrics

To compare US and CT geometries, the similarity between the registered freehand US and CT geometries was measured. First, the US and CT point clouds were resampled to series of equidistant contours in the vessel length direction, i.e., the *y*-direction, with a spacing of circa 0.2 mm depending on the initial sparsity of the US contours. Next, for every *y*-location, the similarity was quantified using the similarity index (SI), also known as Dice coefficient. This is a measure for spatial overlap, defined as

(2) $$SI=\;\frac{2\cdot (P_{US}\;\cap \;P_{CT})}{P_{US}\;+\;P_{CT}}$$

with $P_{US}$ and $P_{CT}$ being the set of pixels present in binary masks generated from US contours and CT contours at each *y*-location. The Hausdorff distance (HD) was used to calculate the maximum of the minimum distances between the registered US contour points *A* = {*a­_1_*, *a­_2_*­, …. *a­_n_*} and CT contour points *B* = {b­_1_, b_­2­_, …. b_n_} of each *y*-location and is defined as

(3) $$d(A,B)=\max\;\left\{\underset{a\in A}{max\;}\underset{b\in B}{min}|b-a|,\underset{b\in B}{max\;}\underset{a\in A}{min}|a-b|\right\}$$

with |*a*–*b*| and |*b*–*a*| being the Euclidean distance between *a* and *b* (28). For each geometry, the SI and the median HD and interquartile range were computed. Furthermore, an overall median SI and HD and interquartile range were computed.

### Mechanical property assessment

Distensibility was computed locally and globally by evaluating area change and volume change respectively and by using the measured pulse pressure data. First the compliance was computed locally, $C_{local}$, and globally, $C_{global}$, according to Equations 4, 5:

(4) $$C_{local}=\;\frac{\Delta A}{\Delta P}$$

(5) $$C_{global}=\;\frac{\Delta V}{\Delta P}$$

where ΔA and ΔV represent the area and volume change between ES and ED, respectively, and ΔP is the pulse pressure. Area change was obtained by converting the ED and ES contours in binary masks, from which the area of the lumen pixels was computed. To extract the volume change, the ED and ES contours were first converted into surface meshes. Next, they were converted into closed solid structures from which the volumes were computed using SpaceClaim software (SpaceClaim, Ansys, 2019 R3). Next, the distensibility was determined locally, $D_{local}$, and globally, $D_{global}$, according to Equations 6, 7:

(6) $$D_{local}=\;\frac{1}{A_{ED}}C_{local}$$

(7) $$D_{global}=\;\frac{1}{V_{ED}}C_{global}$$

where $A_{ED}$ and $V_{ED}$ represent the area and volume at ED respectively. Besides these properties, strain, $\varepsilon_{circ\;}$, was computed locally from the circumference of the aorta at ED and ES according to:

(8) $$\varepsilon_{circ\;}=\;\frac{l_{ES}-l_{ED}}{l_{ED}}$$

where $l_{ES}$ and $l_{ED}$ represent the circumference of the aorta at ES and ED respectively. The circumferences were extracted from the binary masks of the ES and ED contours. For this computation, the aortic tissue was assumed to be incompressible and isotropic, and to have small strains. A Wilcoxon test was performed to test whether distensibility was significant between AAA and healthy aortas (*p*-value <0.05).

## Results

### Geometries

**Figure 2:**
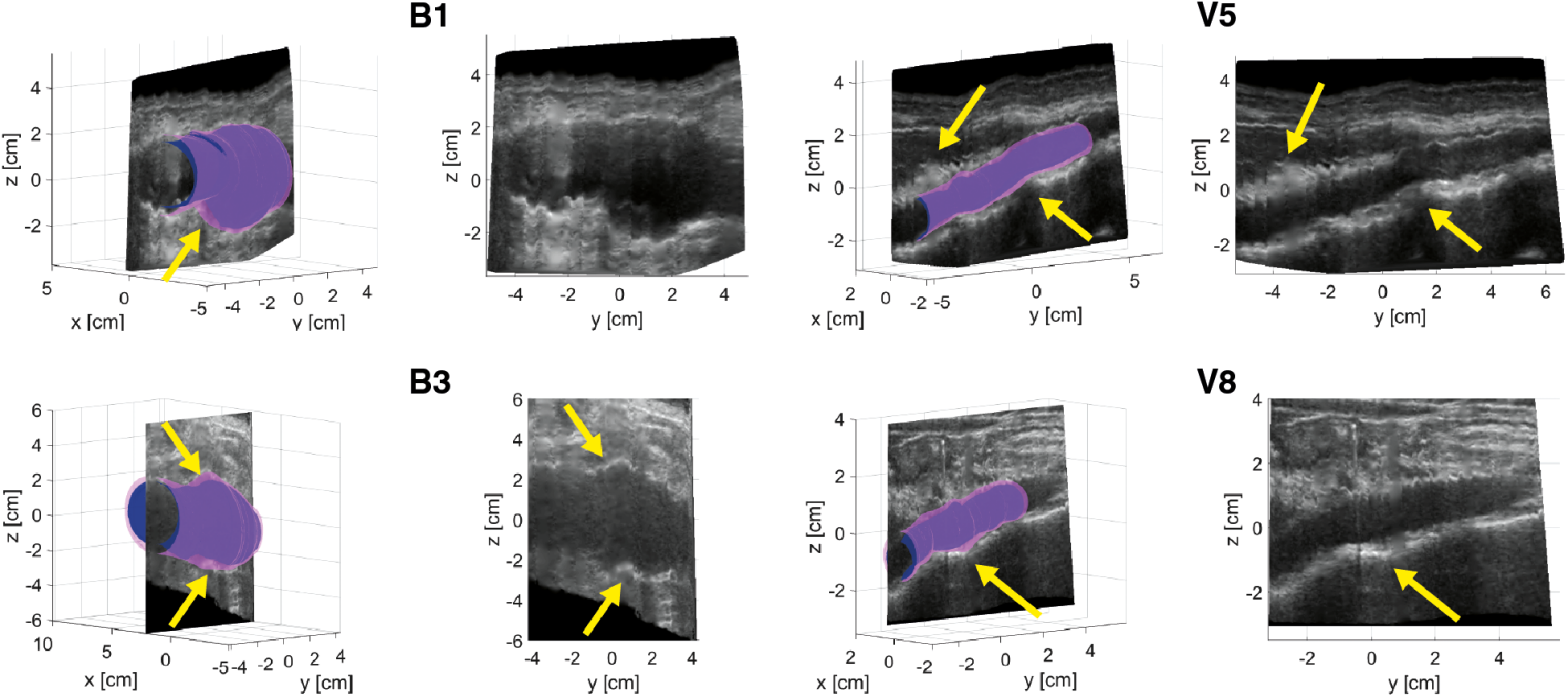
Examples of ED (blue) and ES (purple) geometries of AAAs of patients B1 and B3 and healthy aortas of volunteers V5 and V8. A longitudinal cross-section reconstruction extracted by interpolation of the individual series of US frames is shown separately and together with the geometries. Yellow arrows point at regions of large geometry changes in AAAs and poor image quality regions of healthy aortas.

Figure 2 shows examples of ED and ES geometries assessed from a freehand 2D US dataset of AAA patients B1 and B3 and volunteers V5 and V8 together with a reconstructed cross-section of the US image data showing the vessel throughout the sweep in the longitudinal direction. The distension, present during the acquisition is clearly visible in the US cross-sections. Furthermore, it shows that the vessel dimensions change over the length of the vessel. It can be appreciated that the geometries overall match with the vessel shape. For the AAAs there are regions where a large change in vessel dimension occurs, as indicated with the yellow arrows in Figure 2. Here the ES geometry is more enlarged compared to the ED geometry than in other regions. For the healthy vessels, there are some regions where the wall is less visible due to reduced image quality, indicated by the arrows in Figure 2. In these regions the difference between ES and ED is either strongly increased or strongly decreased compared to the rest of the vessel. The average length of the region that was segmented frame by frame is 60 mm ± 21 (*N* = 10) for volunteers and 65 mm ± 15 for AAA patients (*N* = 16).

**Figure 3:**
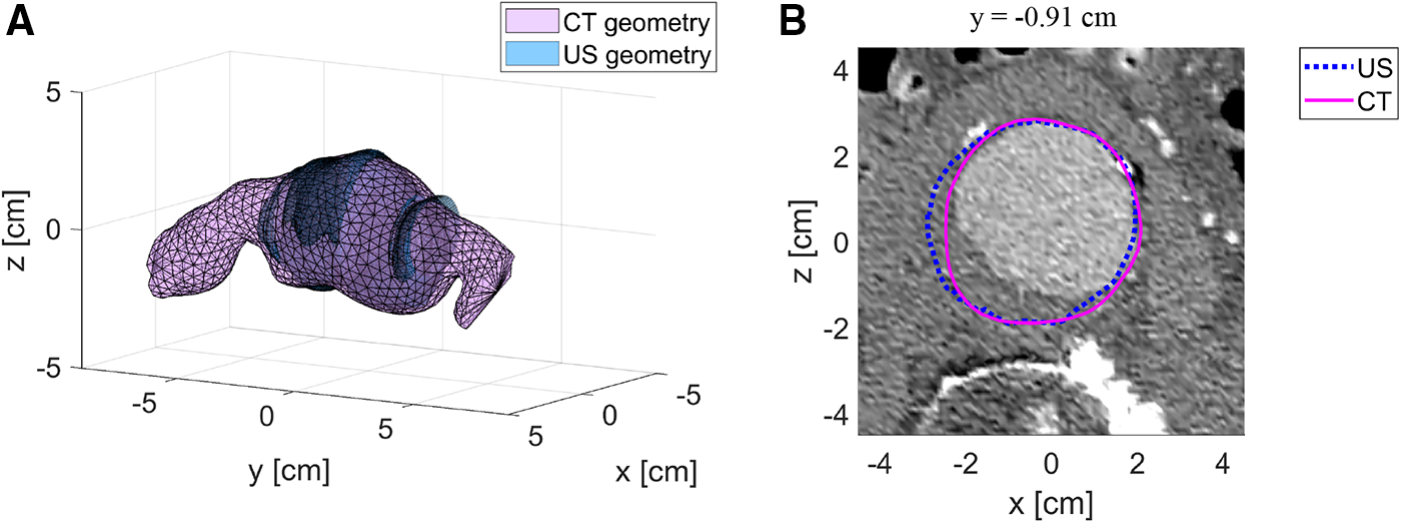
Example of registered US geometry with CT geometry. (**A**) US geometry of patient A2 (blue) with the corresponding CT geometry (purple) after registration. (**B**) Example of a CT image with the US contour (blue) and CT contour (purple) at location *y* = −0.91 cm.

Figure 3A shows the registered CT geometry and US geometry of patient A2 with a similarity index of 0.93. The figure shows that the US geometry generally follows the shape and size of the CT geometry. Furthermore, this example demonstrates that a large field of view can be achieved with freehand 2D US. However, the full aorta including the aneurysm shoulders is not included. In Figure 3B an example is shown of the CT image data together with the AAA wall outline of the CT geometry and US geometry at *y*-location −0.91 cm of Figure 3A. This image shows that deviations are visible at the location where thrombus is present and at the side wall regions. As shown in Table 2, SIs of the nine patients range between 0.88 and 0.93 and the overall median SI and interquartile range is 0.91 [0.90–0.92]. The median HD of the patients are in the range of 3.0 and 7.3 mm, where the overall median HD and interquartile range is 4.7 [3.9–5.6] mm.

### Mechanical properties

**Figure 4:**
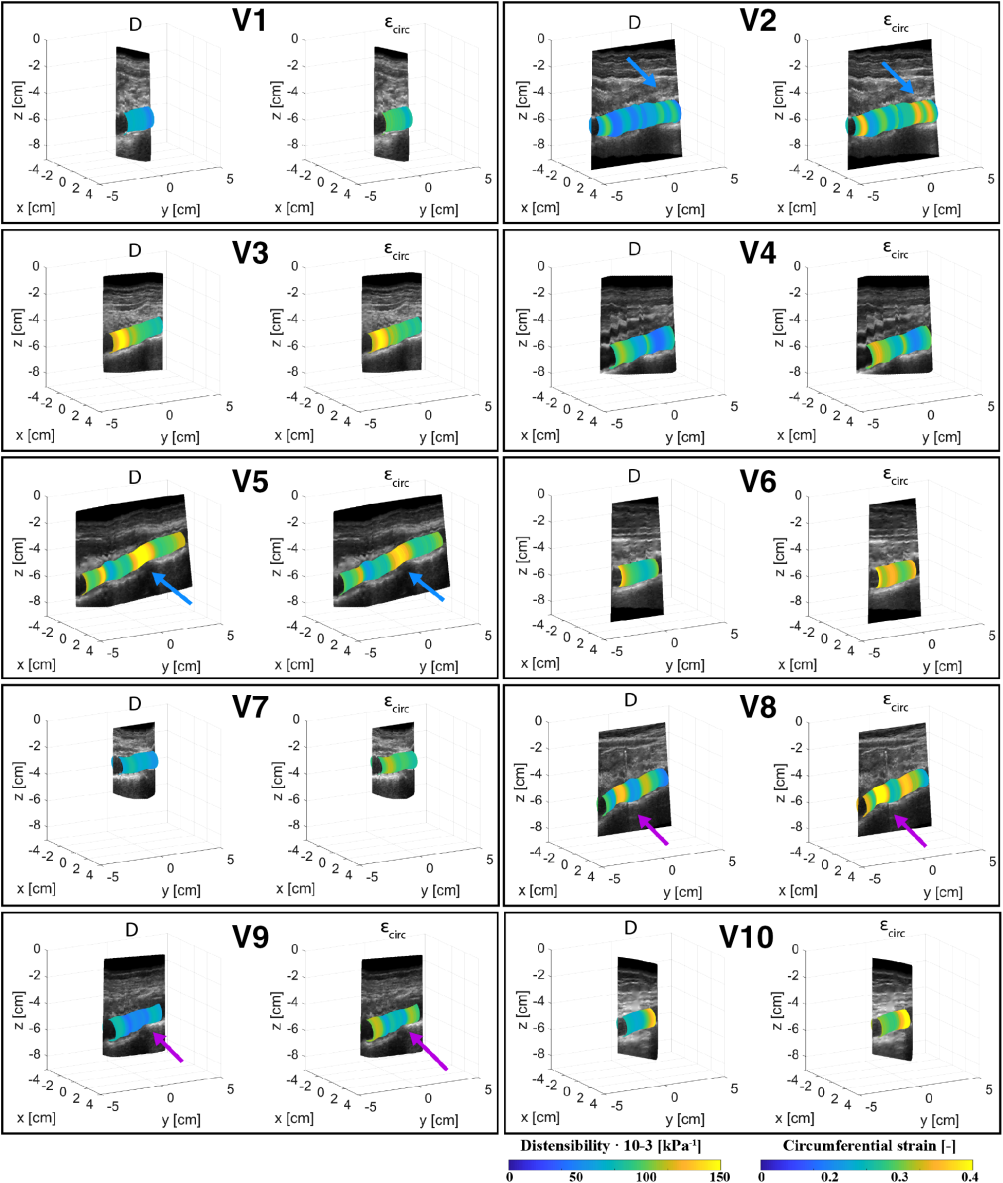
Overview of the assessed mechanical properties distensibility, D·10^−3^ (kPa^−1^), and circumferential strain, $\varepsilon_{circ\;}$ (−), of volunteers V1–V10 mapped onto the mean arterial pressure state geometry and visualized with an interpolated cross-section of the acquired US images. Blue arrows point at locations of poor echogenicity and purple arrows point at regions where the vessel direction changes.

**Figure 5:**
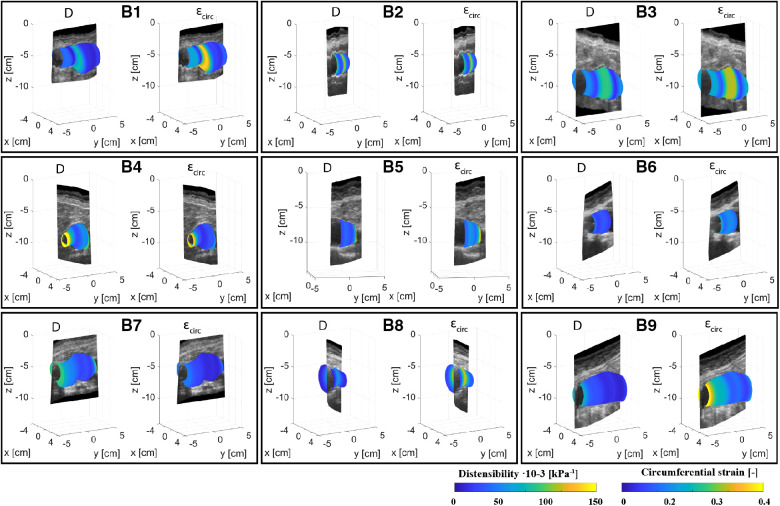
Overview of the assessed mechanical properties distensibility, D·10^−3^ (kPa^−1^), and circumferential strain, $\varepsilon_{circ\;}$ (−), of AAA patients B1–B9 mapped onto the mean arterial pressure state geometry and visualized with an interpolated cross-section of the acquired US images.

Figures 4, 5 show the local distensibility and circumferential strain mapped onto the MAP geometry for healthy volunteers and AAA patients respectively. These are shown together with an interpolated longitudinal cross-section obtained from the acquired US frames. The mean and standard deviation of the local distensibility and strain values are shown in Table 3. The mean distensibility ranges between 60 and 105·10^−3^ kPa^−1^ and 21 and 60·10^−3^ kPa^−1^ for healthy aortas and AAAs respectively. For both healthy aortas and AAAs, variation in distensibility is visible along the length of the vessel. In some volunteers the variation is more gradual (V1, V3, V4, V6, V7, V9, V10), whereas for others the patterns are more scattered (V2, V5, V8). For 7 out of 10 healthy aortas, a decline in distensibility is visible from the proximal to the distal side of the aorta. For volunteers V2 and V5 there are regions where the wall is less visible, as indicated with blue arrows. As shown in Figure 2, the distension is increased in this region for V5. Hence an increase in distensibility is visible in Figure 4. For volunteers V8 and V9, a change of vessel direction is visible, as indicated with the purple arrows. For AAAs, besides the dilated aneurysm region, less dilated regions were also analyzed. In these regions, for patients B4, B5, B6, B7 and B9, the distensibility and strain are larger compared to the dilated region. On the contrary, for patients B1, B3 and B8, an increased distensibility is observed in the dilated region, compared to the less dilated regions (Figure 5). These regions correspond to regions where a large change in vessel shape occurs, as is visible in Figure 2.

**Table 3:** Overview of mean local distensibility, D_local_, and mean local circumferential strain, $\varepsilon_{circ\;}$, of healthy volunteers (V1–V10) and AAA patients (B1–B9).

	Volunteers			AAA patients	
	D_local_·10^−3^ (kPa)	$\varepsilon_{circ\;}$ (−)		D_local_·10^−3^ (kPa)	$\varepsilon_{circ\;}$ (−)
V1	63 ± 7.9	0.23 ± 0.03	B1	27 ± 20	0.13 ± 0.09
V2	65 ± 15	0.24 ± 0.05	B2	59 ± 31	0.16 ± 0.08
V3	105 ± 23	0.28 ± 0.05	B3	45 ± 19	0.16 ± 0.08
V4	77 ± 20	0.24 ± 0.05	B4	42 ± 28	0.18 ± 0.10
V5	104 ± 24	0.25 ± 0.05	B5	60 ± 60	0.22 ± 0.18
V6	90 ± 16	0.30 ± 0.04	B6	27 ± 5.7	0.12 ± 0.02
V7	67 ± 6.5	0.25 ± 0.02	B7	23 ± 12	0.13 ± 0.07
V8	83 ± 20	0.29 ± 0.06	B8	24 ± 15	0.14 ± 0.08
V9	60 ± 13	0.23 ± 0.05	B9	21 ± 13	0.14 ± 0.07
V10	89 ± 27	0.31 ± 0.08			

For each subject, the mean and standard deviation are reported.

**Figure 6:**
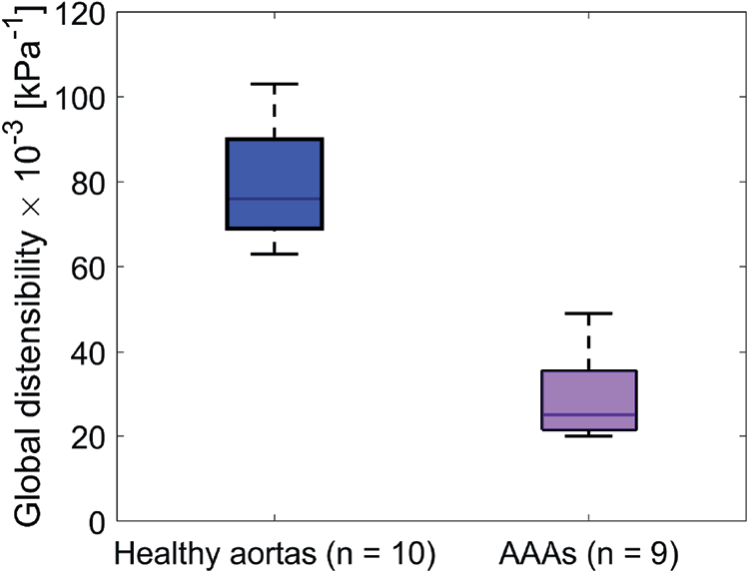
Boxplot of the global distensibility·10^−3^ (kPa^−1^) of healthy aortas (blue) and AAAs (purple).

Global mechanical properties are shown in Table 4. Distensibility of the healthy aortas with a mean and standard deviation of 80 ± 15·10^−3^ kPa^−1^ is larger compared to the AAAs, where the mean and standard deviation is 29 ± 9.6·10^−3^ kPa^−1^. Figure 6 shows that there is a clear distinction between the global distensibility of AAAs compared to healthy aortas. Distensibility of the AAA wall is significantly different from the healthy aorta distensibility (Wilcoxon test: *p*-value = 2·10^−5^).

**Table 4:** Overview of global mechanical properties of healthy volunteers (V1–V10) and AAA patients (B1–B9).

	Volunteers					AAA patients			
	V_ED_ (ml)	V_ES_ (ml)	C_global_ (ml/kPa)	D_global_·10^−3^ (kPa^−3^)		V_ED_ (ml)	V_ES_ (ml)	C_global_ (ml/kPa)	D_global_·10^−3^ (kPa^−1^)
V1	3.65	5.51	0.23	63	B1	76	95	1.9	25
V2	12.2	18.7	0.78	64	B2	40	50	1.0	25
V3	5.16	8.47	0.53	103	B3	101	139	5.0	49
V4	10.3	15.3	0.71	69	B4	33	45	1.2	37
V5	10.4	16.2	1.06	102	B5	24	31	0.8	35
V6	3.75	6.56	0.34	90	B6	30	38	0.8	28
V7	4.12	6.64	0.30	73	B7	92	112	1.8	20
V8	6.37	10.5	0.52	82	B8	18	23	0.4	22
V9	6.67	10.2	0.47	70	B9	95	122	1.9	20
V10	3.09	5.20	0.24	79					
µV	-	-	-	80 ± 15	µB	-	-	-	29 ± 9.6

Global properties V_ED_ (ml), V_ES_ (ml), C_global_ (ml/kPa) and D_global_·10^−3^ (kPa^−1^) are reported.

## Discussion

In this study, healthy abdominal aorta and AAA geometries were assessed from fast probe-tracked freehand 2D US acquisitions, using semi-automatic segmentation and fully automated signal processing. The presence of pulsatility in the acquired data, which typically distorts volumetric image reconstruction, allows for retrieving distensibility both locally by evaluating area change, and globally by evaluating volume change. Furthermore, geometries of AAA patients were compared to CT geometries obtained within 1 month of the US acquisition, showing good similarity. Distensibility was estimated in healthy aortas and AAAs to evaluate whether the approach is sensitive to different and expected levels of distensibility, in both cylindrical and more complex geometries. Moreover, a comparison of the material properties with those found in previous studies was performed.

Using the star-Kalman algorithm, ellipse-shaped contours were detected in each consecutive frame. Although ellipses can closely resemble the aorta shape, they do not fully match with the vessel shape. To achieve this, an additional 2D active contour can be used. These are energy minimizing functions that attract to the lumen-wall or thrombus-wall interface image features and are constrained by internal forces. As with AAAs the diameter changes significantly over the length and AAAs have various shapes and sizes, tuning such an active contour for each frame and for every aorta remains challenging. Furthermore, in the side wall regions, i.e., regions where the radial wall direction is parallel to the lateral axis of the US image, these active contours may fail if not tuned properly. The ellipse approach that was used in our study was robust in images where the image quality at the sides of the aorta was reduced, but the flexibility of the approach is limited, which may lead to errors in some geometries. Therefore, in this study only the ellipse estimate was used. Alternatively, minor manual adaptions could be incorporated to improve the final geometry as was shown in a study by Rouet et al. (29).

Heart frequency detection was used, such that the ED frames could automatically be detected from the area over time signal obtained from the segmented contours. The centers of these frames were then mapped to 3D space and used to generate a centerline. Compared to the study by de Ruijter et al., the centerline was interpolated in 3D space (Figure 1), rather than in the image coordinate system, such that correction for the probe position was considered (20). A single heart frequency was often not distinguishable from the power-density spectrum. Therefore, a frequency range was detected, allowing for small frequency changes when searching for the minima that represent the end diastolic frames. The frequency change throughout the acquisition is likely due to heart frequency changes that commonly occur during breath-hold (30). The acquisitions were performed during breath-hold to limit motion of the aorta within the body due to breathing, which cannot be detected with the probe tracker. When breathing is constant, breathing motion could be filtered out. This would limit the chance to get heart frequency changes throughout the acquisition and it would allow for extended acquisition time.

To obtain estimates of ED and ES geometries, envelope detection was applied on radius-time signals originating from the segmented contours of each frame. The approach relies strongly on the segmentation quality and erroneous radii can lead to inaccuracies in the final geometry. By performing the detection in the *r*-*t­* domain with equidistant timesteps and by constraining peak detection with a minimal peak distance corresponding to the found heart frequency range, erroneous detection of peaks is limited. Figure 2 shows that overall, the geometries correspond well to the US data. However, in regions with large geometry change, which occurs in some AAAs, the ES geometry seems to overestimate the expansion of the vessel, which likely leads to the high distensibility values found in these regions (Figure 5). This could be circumvented by reducing the speed of the probe during the acquisition, such that more heartbeats occur over the length of the vessel. In this way changes due to geometry and changes due to vessel expansion are likely better distinguished such that envelope detection can improve locally. Probe speeds were manually increased for the AAAs, as the time of the breath-hold was commonly shorter for patients, opposed to healthy volunteers. In some datasets, regions with reduced image quality were present. In these regions the difference between the ES geometry and ED geometry was either larger or smaller compared to regions of good image quality, as observed in Figure 2. Furthermore, from Figure 4 it was observed that in these regions the distensibility either strongly increased or decreased compared to other regions.

The MAP AAA geometries obtained were compared to CT geometries by evaluating the similarity and overlap after registration. As shown in Table 2, the median SI and IQ range is 0.91 [0.90–0.92] and the overall median HD and IQ range is 4.7 [3.9–5.6] mm. These values are in the same range as those reported in conventional 3D US studies (7, 29, 31). This demonstrates that the approach proposed for geometry assessment has a high accuracy and is not inferior to 3D US-based approaches, while with this imaging approach additional mechanical properties can locally be assessed using a 2D US system. Visual inspection of the resulting geometries reveals reduced performance in regions where thrombus is present (Figure 3) and where the angle between the US beam direction and the radial direction of the wall is large, i.e., the side wall regions. Hence, as expected, structures in these regions are less clearly visible in US images. Multi-perspective US imaging can help improve the lateral resolution, which can help improve segmentation quality in these regions (32). Registration was performed by iteratively minimizing the distances between the 3D point clouds of the CT and US geometries. Registration of the two datasets is challenging as the AAA is not rigid. Therefore, we limited the time between the US scan and CT scan as much as possible (Table 2). Furthermore, we used the mean arterial pressure state geometry for registration with the CT geometry. In the future, registration can be refined by matching image features.

Distensibility and strain were computed from the assessed ED and ES geometries and the brachial pressure measurements. Local assessments were performed by evaluating area change between ED and ES and global assessment of distensibility was performed by evaluating volume change. As illustrated in Figure 6 and Table 4, global distensibility values were larger for healthy aortas (80 ± 15·10^−3^ kPa^−1^) in comparison with AAAs (29 ± 9.6·10^−3^ kPa^−1^). Compared to previous studies (Table 5), distensibility values of healthy aortas are larger, but are in the same order of magnitude (11, 33). In our study, larger values than in previous literature are likely found, because the healthy volunteers are younger compared to those from previous studies. There is a biological variability in vessel properties over the length of the vessel. A decline in distensibility from the proximal to the distal side of the aorta was visible for 7 out of 10 healthy aortas (Figure 4). Analysis of the local distensibility maps (Figure 4) and visual inspection of the US data and geometries (Figure 2) showed that regions of poor image quality apparently lead to overestimation or underestimation of distensibility. Circumferential strain values of the young healthy adults in our study (0.25 ± 0.03) were comparable to findings by a previous study on young adults (34, 35). Circumferential strains were assessed under the assumptions that the vessels exhibit small strains and that the aortic tissue is incompressible and isotropic, which are simplifications that are often used to model aortas and AAAs (36). Furthermore, the local circumference change is estimated from the spatiotemporal data that was available in the close neighborhood. In the future, higher frame rates and improved image quality can potentially lead to a more local approach where the local heterogeneity in the circumference of the wall could be studied, by using methods such as speckle tracking (34, 35).

**Table 5:** Comparison of our distensibility findings with other studies.

Study	System	Distension assessment	Distensibility·10^−3^ (kPa^−1^)	AAA diameter (mm)	Age mean (±SD)
Our study	Freehand 2D US	Volume; healthy	80 ± 15	-	26 ± 2
		Volume; AAAs	29 ± 9.6	47 ± 6	75 ± 9
		Area; healthy; local	60–105 (range)	-	26 ± 2
		Area; AAA; local	21–60 (range)	47 ± 6	75 ± 9
Long et al. (2004a)	Tissue Doppler	Mean segmental diameter; healthy	37 ± 13	-	34 ± 10
Long et al. (2004b)	Tissue Doppler	Mean segmental diameter; AAAs	6.1 ± 3.6	39 ± 9	70 ± 7.6
Rose et al. (2010)	Cine MR	Area; healthy	50 ± 17	-	29 ± 4
Zha et al. (2017)	CT	Area; below renal artery; AAAs	10.5 ± 2.2	>3.0 cm	67.2 ± 6.8
		Area; at max diameter; AAAs	4.9 ± 1.8		
Van ‘t Veer et al. (2008)	MRI	Volume, AAAs	2 ± 0.5	58 ± 6.0	73.6 ± 6.4
Molacek et al. (2011)	CT	Area; AAA region	3.7–56 (range)	60 ± 16	65
		Area; AAA Non-dilated region	12–42 (range)		

As illustrated in Figure 5, distensibility was for most AAA cases smaller in dilated vessel regions compared to the non-dilated, or less dilated regions, which is in accordance with previous studies (37–39). As shown in Table 5 distensibility values of the AAA patients fell within the range of a study by Molacek et al. but were larger than those obtained in other studies (9, 12, 38, 39). Although the geometries match well with the gold standard, segmentation errors may lead to over or underestimation of distensibility. Commonly, diameter-based distension values are computed from US data for distensibility computation. The compliance of the vessel is then often computed with the assumption that an increase in diameter leads to an area change that is twice as big (11, 12). This has been derived for circular shapes (40). However, AAAs are typically non-circular and healthy abdominal aortas are not necessarily a circle (41). Hence this assumption is not fully reliable. Evaluation of area change, or volume change would allow for accurate compliance assessment. US imaging is considered safer than MRI or CT. However, with US imaging the image is generally reduced in the side wall regions compared to the upper and lower wall regions, due to the physics of US. This can lead to inaccuracies in area or volume measurements. Multiperspective US imaging or artificial intelligence techniques that can optimize the image quality could further improve the reliability of compliance assessment from lumen area change as opposed to a diameter-based approach.

Freehand 2D US acquisition is fast and save and can easily be performed in the clinical workflow. In addition to this, the offline segmentation of the vessel wall is semi-automatic, and the remainder of the workflow is fully automatic, allowing for quick analysis. With the approach we propose, we can obtain both patient-specific geometries and mechanical properties such as distensibility and circumferential strain. Locally we could estimate the distension of the vessel based on automatically detected sample points in the end diastolic and end systolic phase of the cardiac cycle. The spatial resolution, temporal resolution and the field-of-view that can be achieved with freehand 2D US clearly outperform those provided by conventional 3D US imaging. Hence, this approach can provide more complete data (i.e., full geometry, local material properties) for making a personalized finite element model of the aneurysm, as was previously performed with conventional 3D US imaging (6), but with a simpler 2D US device. Furthermore, application may now be considered for AAA follow-up studies. These studies can help to determine how these biomechanical parameters relate to AAA growth and AAA rupture risk. Distensibility assessment from freehand 2D US is not limited to wall distension only and could be extended for studying distension of the lumen-thrombus interface as well.

A limitation of the imaging approach in this study is the fixed sampling rate of 25 Hz of the acquisition system combined with the probe tracker. As the probe is moved freehand, the distance between frames may vary within one acquisition and between acquisitions of different subjects. It is expected that local estimations of distensibility are more accurate in regions where the samples are closer together, i.e., with slower probe speeds. Specifically in regions where besides changes due to pulsatility, the vessel dimension changes significantly within one heartbeat, unexpected values of distensibility were observed. There is a tradeoff between field-of-view and sample density. A too slow probe speed will reduce patient comfort. Moreover, the image quality may be hampered by patient body motion and motions due to breathing. In the future, instructing and training sonographers to perform the acquisition with reasonable speeds can help to reduce the geometrical spacing between frames as much as possible, while maintaining a large field-of-view.

What remains challenging with freehand 2D US is assessment of the full geometry. The average length of the captured region that was segmented was 60 mm ± 21 for volunteers and 65 mm ± 15 for AAA patients. Due to bowel gas and obesity, the image quality can locally be reduced, leading to incomplete measurements, as has previously been reported in studies with freehand 2D US (14, 15, 17). However, similar challenges occur with conventional 2D US imaging or 3D US imaging with a matrix array. The focus of this study was to evaluate quality of geometry assessment compared to CT and to demonstrate assessment of distensibility based on area change and volume change. Therefore, datasets that included a part of the region of interest were still included and only datasets with poor image quality throughout the entire acquisition were discarded. Acquisitions are performed during breath-hold, which limits acquisition time and thereby the field of view. To increase the field of view, multiple acquisitions could be performed, where the best data of each acquisition could be included and registered. Alternatively, acquisitions could be performed without breath-hold. Besides this, additional training for freehand 2D US imaging could help to improve image quality and field of view.

Freehand 2D US acquisition is fast and save and can easily be performed in the clinical workflow. In addition to this, the offline segmentation of the lumen-wall or thrombus-wall interface is semi-automatic, and the remainder of the workflow is fully automatic, allowing for quick analysis. Distensibility assessment from freehand 2D US is not limited to wall distension only and could be extended for studying distension of the lumen-thrombus interface, which could help to further model and characterize AAAs.

## Conclusion

To conclude, in this study we propose a novel approach that uses the pulsatility that is captured with freehand 2D US imaging for distensibility assessment of AAAs. Where pulsatility typically hampers reconstruction of volumetric images and is therefore neglected, it can be utilized to retrieve additional information from the dataset besides geometries. Registration and comparison with CT showed good overall overlap between geometries. Furthermore, results are comparable to studies that assessed geometries from 3D US data obtained with a matrix probe, while the higher frame rate of freehand 2D US combined with signal processing allows for local distensibility assessment. The method performs as expected in regions with sufficient image quality but needs improvements for regions with large geometry changes and poor image quality. This could be mitigated by reducing the probe speed, advanced motion filtering of motions due to breathing, and by performing multi-perspective ultrasound imaging. In the future, the approach can be further expanded with quantification of distension of the lumen-thrombus interface.

## Data Availability

The raw data supporting the conclusions of this article will be made available by the authors, without undue reservation.
